# Supplementary material for: Health Status and Access to Healthcare for Uninsured Migrants in Germany: A Qualitative Study on the Involvement of Public Authorities in Nine Cities
Source: Int J Environ Res Public Health. 2022 May 28;19(11):6613. doi: 10.3390/ijerph19116613 (PMC9180213; doi:10.3390/ijerph19116613)
Supplement: Supplementary file 1 [file ijerph-19-06613-s001.zip › Supplementary File S1 Interview Guide German and English version 11-04-2022.pdf]

Supplementary File S1 : Interview guide German original and English courtesy translation, reproduced for publication. (Version 2, 07/2017)

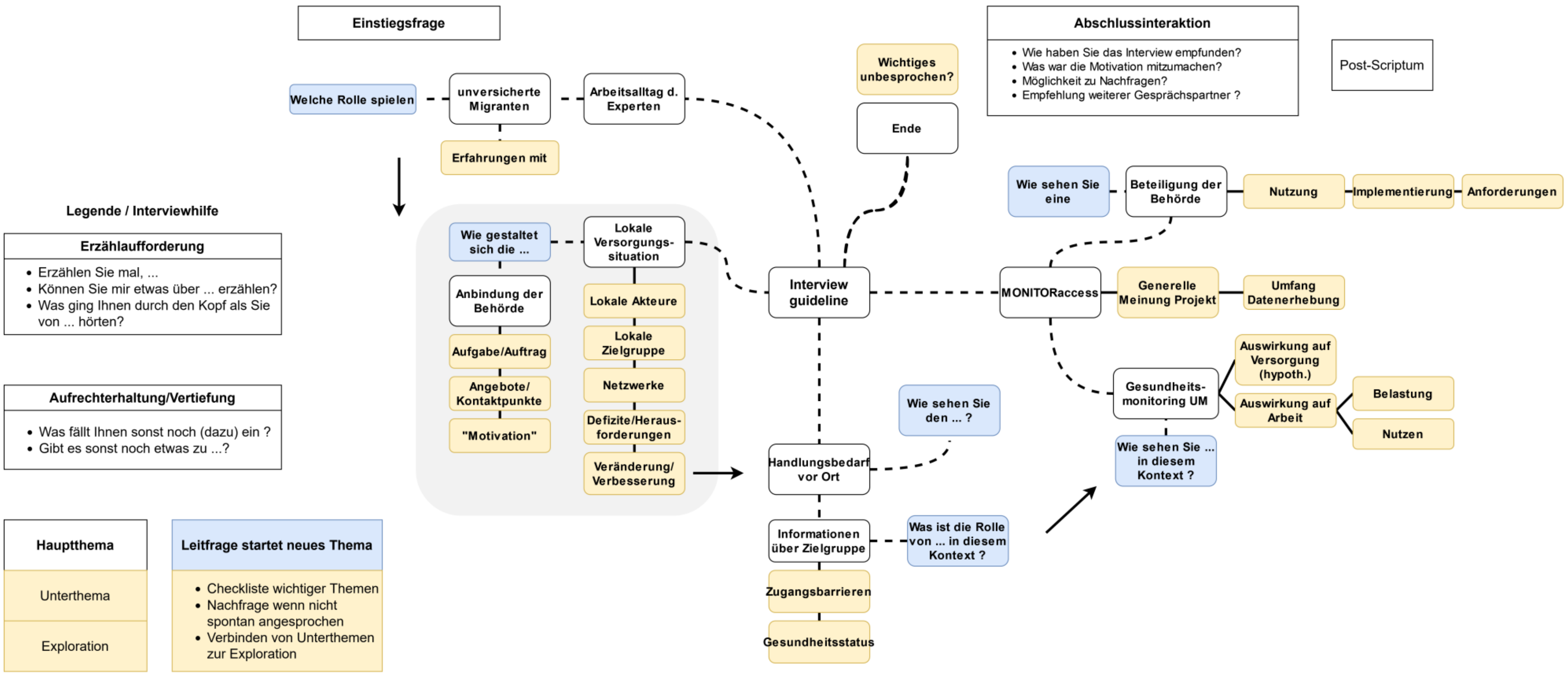

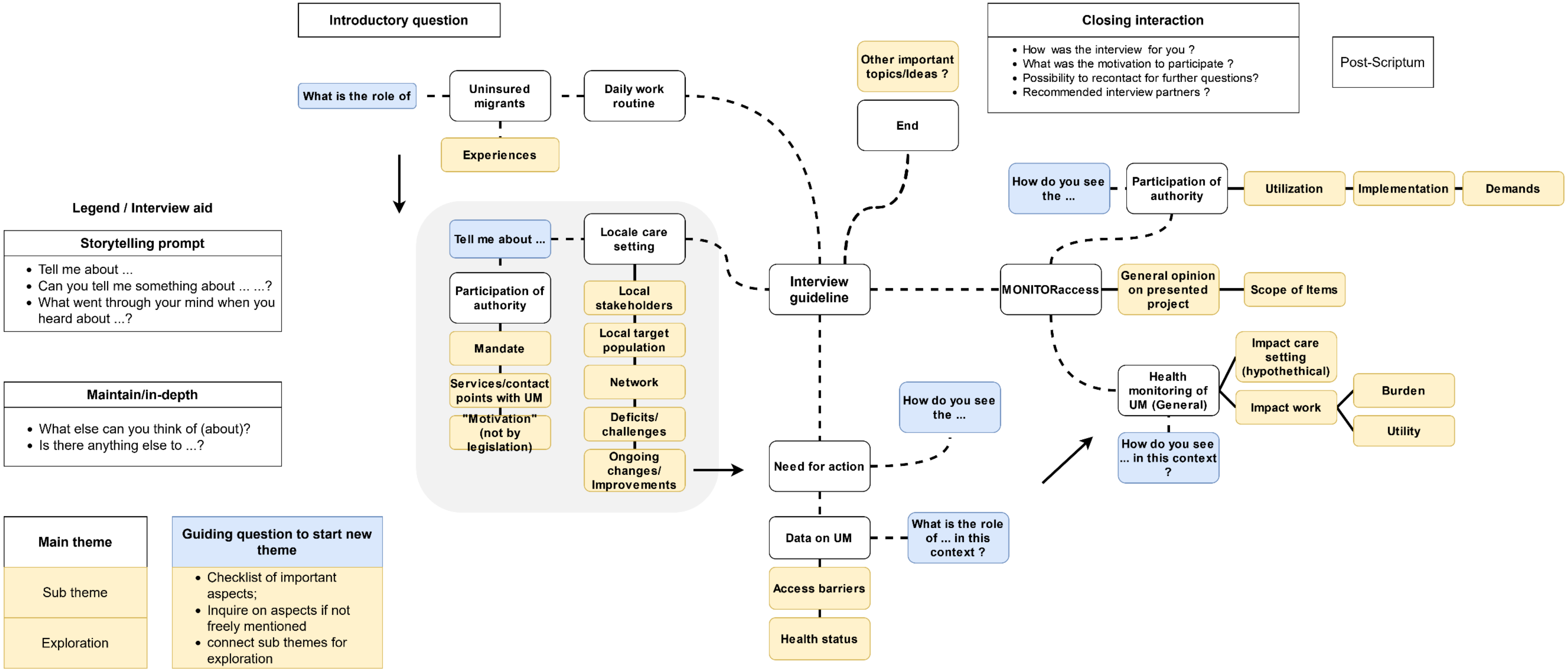

Introductory question
What is the role of
Uninsured migrants
Daily work routine
Experiences
Legend / Interview aid
Storytelling prompt
• Tell me about ...
• Can you tell me something about ... ...?
• What went through your mind when you heard about ...?
Maintain/in-depth
• What else can you think of (about)?
• Is there anything else to ...?
Main theme
Sub theme
Exploration
Guiding question to start new theme
• Checklist of important aspects;
• Inquire on aspects if not freely mentioned
• connect sub themes for exploration
Tell me about ...
Locale care setting
Participation of authority
Local stakeholders
Mandate
Local target population
Services/contact points with UM
Network
"Motivation" (not by legislation)
Deficits/ challenges
Ongoing changes/ Improvements
Other important topics/Ideas ?
End
Interview guideline
Need for action
How do you see the ...
Data on UM
What is the role of ... in this context ?
Access barriers
Health status
Closing interaction
• How was the interview for you ?
• What was the motivation to participate ?
• Possibility to recontact for further questions?
• Recommended interview partners ?
Post-Scriptum
How do you see the ...
Participation of authority
Utilization
Implementation
Demands
MONITORaccess
General opinion on presented project
Scope of Items
Health monitoring of UM (General)
Impact care setting (hypothethical)
Impact work
Burden
Utility
How do you see ... in this context ?
