# Supplementary material for: Health Status and Access to Healthcare for Uninsured Migrants in Germany: A Qualitative Study on the Involvement of Public Authorities in Nine Cities
Source: Int J Environ Res Public Health. 2022 May 28;19(11):6613. doi: 10.3390/ijerph19116613 (PMC9180213; doi:10.3390/ijerph19116613)
Supplement: Supplementary file 1 [file ijerph-19-06613-s001.zip › Supplementary File S2_Data table, illustrating the reductive content analysis and the inductive-deductive code framework .pdf]

Supplementary File S2 : Data table, illustrating the reductive content analysis

Abbreviations and explanation

Document	File name of the audio file transcript
Start	Start of quote in the audio file transcript
Code	Code name of the quote
End	End of the quote in the audio file transcript
Segment	Quoted segment
SUMMARIZATION	Short summarization of the quote to expose the analyzed content according to the applied code
CASE	Commentary on the analyzation on case level
INTERCASE	Commentary on the analyzation on comparison between cases in the sample
ANNOTATION	Information that were researched and added for the analysis when contet was not clear from the interview. We can not expose these sources as they enable the recognition of individual public authorities and exper
Capitalized Themes	Main topics in capital letters have been noted in the SUMMARIZATION column for better finding
LPHA	Local publich health authority/authorities
SSO	Social security office/offices
NGO(s)	Non-governmental organization(s)
P1, P2 etc	Interviewpartner 1, Interviewpartner 2; In interview 7 we have named the two interviewpartners P71 and P72
Codes:	We have noted a summary of the code framework on Spread sheet 2 "Code Framework"
BLANK	We have blanked out certain excerpts to preserve anonymity of authorities and experts for this publication

Exemplary courtesy translation of 5 segments from the original data table for illustration of research process

Document	Start	Code	End	Segment	SUMMARIZATION	CASE	INTERCASE
**Interview Audio 04**	6	02 ANBINDUNG BEHOERDE (INVOLVEMENT OF AUTHORITY)	6	The authority here as such, i.e. a social authority itself, the topic is actually only located here in our department. Apart from that, we have health assistance for social welfare recipients, we have health management for asylum seekers. Otherwise, such topics, including people without health insurance, access to health care, health insurance law, are all located in the areas of the health authority. We are a bit of a lone wolf here - as far as health issues are concerned, I'd say.	The topic is a marginal topic in the social authority	Ultimately, the topic is only explicitly located with the expert	Smilar to P11
**Interview Audio 02**	8	02 ANBINDUNG BEHOERDE (INVOLVEMENT OF AUTHORITY)\" Runder Tisch" ("Round table")	8	Well, for about a year now I've wanted to have a round table with all the hospitals, with the youth welfare office and with the outpatient providers of care for people without health insurance. For people without health insurance. It is a very political topic, when I started my job one and a half years ago, I first interviewed all 16 hospitals in [town]. The social workers, the patient administration and, if they had time, the management. What are the problems? From the different.. social workers see it differently than the patient administrators and the and billing and exactly what kind of difficulties there are, how long hospitalisation there are, how long the waiting times are, how much there are costs that they are stuck with, what the difficulties are and qualitative examples. And I summarised that in a short report, which was - yes, change of department etc. long political - yes, it was then sent around and I am actually waiting, yes, we have been trying to set up a round table since the beginning of these years. It's just taking a while until it can be set up now	NETWORK : Round Table : The expert is in the process of setting up a "round table" with representatives of all hospitals, the youth and social and social services and with the outpatient providers of care for people without health insurance. The occasion is her survey of hospitals on the challenge of inpatient care for uninsured patients.	Inpatient needs are a challenge according to the expert. Planned participants : Representatives of all hospitals, social services, outpatient outpatient contact points	Inpatient needs are generally a more frequently mentioned challenge. cf VERSORGUNGSSITUATION (CARE SITUATION)
**Interview Audio 02**	8	02 ANBINDUNG BEHOERDE (INVOLVEMENT OF AUTHORITY)\" Runder Tisch" ("Round table")	8	So that was another initiative at the outpatient level, the other one more inpatient with the round table	NETWORK : round table : The round table refers to inpatient needs of uninsured people.		Round table mentioned in several cases, always interdisciplinary and inter-institutional, concerns and institutions represented vary (NGOs-LPHA-SSO)
**Interview Audio 06**	8	02 ANBINDUNG BEHOERDE (INVOLVEMENT OF AUTHORITY)\" Runder Tisch" ("Round table")	8	The South Eastern Europe Working Group, which is based here in the city administration, is also made up of various specialist areas and external cooperation partners. It has to do with the fact that [the town] has a very high proportion of South-Eastern European immigrants, which is really very high, and they are very often not insured. But they are also a difficult target group, so even when their needs are identified, they suddenly disappear again. Which then of course raises the issue of measures and what resources you then put in to do something for the group is a bit difficult, because you can never really plan it.	Round table of health authority, NGO, regulatory authorities (ANNOTATION 4)	Focus on EU citizens in the the working group; The high mobility of the target group is mentioned ("difficult, planning uncertainty")"	The high mobility in reality is also mentioned by other experts, the working group is problem-oriented (health/social/legal) vs "pluri-local" realities of life (Eichler 2008).
**Interview Audio 06**	40	02 ANBINDUNG BEHOERDE (INVOLVEMENT OF AUTHORITY)\" Runder Tisch" ("Round table")	40	Because if I remember correctly, we had a meeting some time ago with the health conference in the steering committee and our health policy spokespersons. That is, from the parliamentary groups in the municipal council. This is a round table that we want to establish on a regular basis so that we can bring together experts and politicians, so to speak, because we actually work together and they need to know what makes them tick and what is going on with us and what the state of affairs on our side	NETWORK: Round Table: Here is an example of a composition of administrative experts and politicians.	"We actually work together and they need to know what makes them tick and what is going on with us and what are the state of affairs with us" Conversely, there tends	Cf. P10 and P11: active exchange with decision-makers, at least example of project drafts being proactively drafted by public authorities

CODE	DESCRIPTION	INDUCTIV	DEDUCTIV
**02 ANBINDUNG BEHOERDE**	Alle Aussagen bzgl. Einer Anbindung der Behörde an die Versorgungssituation (Direkt, indirekt, Netzwerke).	x	
"Runder Tisch"	Erwähnung von Gremium oder Treffen mit Regelmässigkeit von Akteuren. Mehrere Behörden, oder mit NGO oder anderen Akteuren des Parallelsystems. Zum Thema Unversicherte Migranten.		x
Restrictive Gatekeeper	Zugang limitieren, "Missbrauch" verhindern<br>Gatekeeper im Sinne von es bestehen Ressourcen die genutzt werden können, jedoch entscheidet der Gatekeeper darüber wer einen Zugang erhält oder nicht. Hier wurde codiert wenn der Zugang limitiert oder verwehrt wird. Deduktiv		x
Pull-Faktor	Benennug von Pull-Faktor zur limitierung von Zugänglichkeit. Deduktiv		x
**02.1 EIGENE SERVICES**	Services am Amt, von Amtswegen her; nicht in Trägerschaft	x	
Eigenes Psycho-soz Angebot	Beschreibung von Angeboten mit psychosozialem Schwerpunkt, z.B. Sozialpsychiatrischer Dienst		x
Fortbildung	Benennung zum Aspekt "Fortbildung" im Zusammenhang mit der Arbeit im Parallelsystem (medizinisch und soz rechtlich)		x
Aufsuchende Artbeit	Aussagen zu aufsuchender Arbeit		x
Finanzierung/Steuerung	Benennung von Zuwendungsfinanzierungen, Fond Lösungen und anderen Finanzierungen durch die Behörde, mit öffentlichen Geldern		x
Eigenes Verw Recht Angebot	Arbeit in der Behörde die sozial verwaltungstechnische Fragestellungen der Zielgruppe bearbeiten<br>- DIFF zu Soz rectlich : Soz Rechtlich im Sinne als Service für die beratende Person, Sozial verwaltungstechnisch im Sinne für die Verwaltung und evtl. gegen das Interesse der Person		x
Eigenes Soz Recht Angebot	Angebote, Beratungsstellen, in der Behörde die sozial rechtliche Fragestellungen der Zielgruppe bearbeiten<br>- DIFF zu Soz Verwaltungstechnisch : Soz Rechtlich im Sinne als Service für die beratende Person, Sozial verwaltungstechnisch im Sinne für die Verwaltung und evtl. gegen das Interesse der Person		x
Socio legal Clearing	Clearing, Clearingstelle, tiefgreifende sozialrechtliche Beratung und Vertretung der Klientel zum Erreichen einer Krankenversicherung		x
Couns Pregnancy	Schwangerschaftsberatung, Schwangerschaftskonfliktberatung		x
Couns IfSG	Beratung im Rahmen des Infektionsschutzgesetzes, Beratung zu Infektionserkrankungen		x
Eigenes Mediz Angebot	Bennennung von medizinischen Angeboten/Anlaufstellen die direkt am Amt angesiedelt sind und keine externe Trägerschaft haben		x
Birth Programm	Geburtenprogramme, primär Absicherung der Geburt, sekundär Schwangerschaftsbetreuung		x
Treatm Allgemein	Behandlung von allgemeinen Erkrankungen, nicht nur IfSG		x
Treatm IfSG	Behandlung von Erkrankungen i.R. des IfSG		x
Diagn Sexualhealth/Gyn	Diagnostik im Kontext sexuelle Gesundheit und Gynäkologie, inkl. Schwangerschaftsbetreuung		x
Diagn IfSG	Diagnostik i.R. des IfSG		x
**02.2 EXPERTENARBEIT**	Aufgabe des Experten der den Kontakt zu UM herstellt, direkt und indirekt, in der behördlichen Aktivität	x	
Rolle UM im Alltag	Direkter Kontakt mit UM im Alltag, Kontakt mit dem Thema UM im Alltag (z.B. Koordination von Projekten mit UM, Gremiem zum Thema UM, Datenarbeit)	x	
Mitarbeit in NGO	Freistellung eines Mitarbeiters zum praktischen Arbeiten in einer NGO; Mitarbeit in einer NGO als Teil der behördlichen Aufgabe		x
Laufbahn	Aussagen zu der eigenen Laufbahn<br>- Erfahrungen aus vorheriger Arbeit<br>- Qualifikationen	x	
**02.3 NETZWERK**	Indirekte Teilnahme an der Versorgung über Gremien, runde Tische<br>- Bennennung Netzwerk, Zusammenarbeit, Gremien, runde Tische<br>- Beschreibung der Zusammenarbeit	x	
Interakt Zivil gesellschaftlich	Interaktion mit NGO, Interessengruppen aus der Bevölkerung, Lobby Gruppen für UM	x	
Incoming	Interaktion die nicht Behörde auf NGO auslagern von Services (Subsidaritätsprinzip) ist.<br>- Gesprächsrunde/Runder Tisch ist eigener Code<br>- z.B. "Zusammenarbeit"		x
Outsourcing (Finanziert)	Aussagen zu Akteuren im Parallelsystem die vollumfänglich bezahlt tätig werden (Trägerschaft)		x
Outsourcing (Ehrenamt)	Aussagen zu Akteuren im Parallelsystem die ehrenamtlich/kostenlos oder teilkompensiert tätig werden		x
Interakt Gesundheitssystem	Inkl. Krankenhäuser, Praxen, ohne direkte Anbindung an die Behörde<br>- Ehrenamtliche Arbeit<br>- Kostenvergünstigte Uebernahme von Behandlungen<br>- Rechnungsstellung		x
Krankenhäuser	Benennung von Krankenhäusern im Parallelsystme	x	
Fachärzte	Benenung von Fachärzten im Parallelsystem		x
Interakt Politik	Interaktionen mit Entscheidungsträgern		x

Arbeit mit Politik	Arbeit für Politik, z.B. Beschlüsse schreiben, Sachstandsberichte		x
Auftrag	Aussagen zum Auftrag der behördlichen Arbeit (rechtlich, hierarchisch)	x	
Interakt Behörde, andere	Anbindung mit anderen Behörden	x	
Konflikt	Konflikte in der Zusammenarbeit mit anderen Behörden		x
Kooperativ	Kooperation/Kooperative Zusammenarbeit mit anderen Behörden		x
**3 VERSORGUNGSSITUATION**	Alle Aussagen bzgl. der gesundheitlichen Versorgungssituation von unversicherten Migranten lokal und deutschlandweit, Gesundheitsstatus und Zugangsbarrieren	x	
Prävention			x
Impfen	Aussagen zu Impfungen im Parallelsystem		x
Krankheit			x
Infektionskrankheit	Aussagen zu Infektionskrankheiten<br>- Hierzu zählen alle Angebote im Rahmen des IfSG		x
Hepatitis	Aussagen zu Hepatitis		x
HIV	Aussagen zu HIV		x
Tbc	Aussagen zu Tuberkulose		x
STI	Aussagen zu anderen STI		x
Stationäre Bedarfe/Teure Bedarfe	Benennung von stationären Bedarfen<br>- Beschreibung des Umgangs mit stationären Bedarfen		x
Ambulante Bedarfe	Beschreibung von Ambulanten Bedarfen<br>- Benennung, Aspekte des Umgangs mit diesen Bedarfen		x
Gynäkologie	Aussagen zu Frauengesundheit<br>- reproduktive Gesundheit<br>- Abtreibungen<br>- Krebsvorsorge<br><br>Benennung von Versorgern von Frauengesundheit<br><br>Nicht STI, HIV, TBc		x
Chronische Erkrankungen	Aussagen zu chronischen Erkrankungen im Versorgungssetting<br>- inkl. Infektionskrankheiten die langfristige Behandlungen brauchen<br>- inkl. nicht übertragbare Erkrankungen (z.B. Diabetes)		x
Onkologische Erkrankung	Aussagen zu onkologischen Erkrankungen<br>" Krebs", "Myome"		x
Psychologisch	Aussagen zu Psychosozialen Angeboten<br>- Sozialpsychiatrischer Dienst<br>- Psychische Gesundheit<br>- Diff zu Suchterkrankungen als eigene Categorie		x
Suchterkrankung	Aussagen zu Sucherkrankten und Menschen/Migranten mit Suchterkrankung<br>Aussagen zu Suchterkrankungen im Versorgungssystem		x
Herausforderung/Limit Status Quo	Beispiel einer nicht lösbaren Aufgabe, reell/hypothetisch<br>Nennung von Herausforderungen in der Arbeit der gesundheitsversorgung von unversicherten Migranten, inkl. sozial rechtliche Aspekte<br>Nennung von Herausfoderungen für die Zielgruppe bzgl. gesundheitlichen Bedarfen<br>Nennung von persönlichen Herausforderungen für die Experten	x	
Nichtregierungs Organisation	Aussagen zu NGO Tätigkeiten im Versorgungssetting<br>- Interaktion mit NGOs im Versorgungssetting<br>- Nennung von NGOs im Versorgungssettung		x
Versorgungssetting vor Ort	Beschreibung der VS vor Ort	x	
"Bedingt positiv"	Beschreibung der Versorgungssituation vor Ort als "bedingt positiv" oder teilweise ausreichend oder eine andere Aussage zu einem teilkompensierten Care Setting aus Sicht der Experten		x
"Makeshift Solutions"	Lösungen zu Herausforderungen durch Behilfskonstrukte, z.B. Remigration statt Inanspruchnahme von Leistungen in Deutschland. "Makeshift" Lösungen funktionieren nur im Einzelfall, sind keine langfristige Lösung.		x
Entwicklung VS	Beschreibung eines Vorgangs der zu der Angebotsentwicklung beigetragen hat<br>- Wunsch oder Vorhaben einer Entwicklung des Angebots<br>- Dynamiken/Anpassung in der Vergangenheit zur Entwicklung des Angebots		x
Clearing	Alle Aussagen zu Clearingstellen im Sinne einer sozialrechtlichen Klärungsstelle für unversicherte, nicht in Bezug auf einen EIGENEN SERVICE<br>- Aussagen zum Clearing als sozialrechtliche Klärung eines möglichen Zugangs zum Regelsystem, auch wenn keine spezifische Clearingstelle beteiligt ist		x
Gesundheitsstatus	Aussagen zu Gesundheitsstatus der Zielgruppe	x	

Zugangsbarrieren	Aussagen zu Zugangsbarrieren der Zielgruppe zu medizinischen Hilfen<br>- im Parallelsystem, sowie auch Regelsystem<br>- Beschreibung von Annahmen zu Zugangsbarrieren in Behörden für die Zielgruppe	x	
Institutionell			x
Rechtlich			x
Oekonomisch			x
**4 MONITORING**	Alle Aussagen bzgl. Daten und Datenarbeit im Zusammenhang mit unversicherten Migranten (Behörde, andere Stellen, deutschlandweit)	x	
Grundeinschätzung	Allgemeine äusserungen zum Monitoring, positive oder negative Meinung<br>- Sammelcode für nicht weiter spezifizierte äusserungen	x	
Implementierung	Alle Aussagen zu Anforderungen, Erfahrungen und Empfehlungen bzgl. eines Versuchs einer Implementierung eines Monitorings	x	
Umsetzbarkeit	Aussagen zur Einschätzung wie eine Implementierung sein müsste, d.h. inhaltiche und institutionelle Anforderungen.<br><br>Diff zu Einsetzbarkeit : Dies sind Aussagen die das WO einer Implementierung betreffen	x	
Einsatzbarkeit	Aussagen zur Einschätzung wo und ob eine Erhebung in der befragten Behörde Sinn macht, oder Aussage zu Einschätzung WO noch es sinnvoll wäre<br><br>Diff zu Umsetzbarkeit : Dies sind Aussagen die das WIE einer Implementierung betreffen<br>- z.B. Anregungen geben was berücksichtigt werden muss, jedoch nicht nur den Einsatzort benennen.	x	
Item	Aussage zu einem speziellen Item auf dem Erhebungsbogen<br>Ersetzt den Code Einsetzbarkeit und Umfang wenn diese sich auf speziell ein Item beziehen<br>Aussagen zu einem fehlenden Item	x	
Unique Identifier	: Aussagen zu einem Unique Identifier System, zu der Nachverfolgbarkeit einer Person im Parallelsystem		x
Verwendung	Aussagen zu Verwendung eines Monitorings<br>- reale und hypotetische	x	
Lobbying	Code : Behörde als Lobbying Organ		x
Wissen	Sammelcode zu Aussagen zu aktuellemen Wissenstand zur Zielgruppe durch statistische Erfassung<br>- Wissen von anderen organisationen, auch Fallbeispiele<br>- Eigene Datenerhebung<br>- Andere Berichterstattung<br>- Wissenslücken	x	
Wissensbenennung Inhalt	Inhaltiche Aussagen aus einer Datenerhebung in der Behörde oder einer anderen Quelle<br>- Benennung von Wissenslücken	x	
Wissenslücke	Benennung von expliziten Wissenslücken		x
Art Wissensquelle (quanti/qual)	Benennung von dem Wissenstypus qualitative oder quantitative Daten in Bezug auf eine Wissensquelle		x
Wissensquelle in der Behörde	Aussagen zu Datenerhebungen in der Behörde oder durch behördliche Arbeit	x	
Wissensquelle andere als Behörde	Benennung von Wissensquellen ausserhalb der Behörde (z.B. NGO)		x
Berichterstattung	Berichterstattung der Behörde<br>- Berichte für Politik, Sachstandsberichte, Verwendungsnachweise, wissenschaftliche Publikationen	x	
