# Supplementary material for: Health Status and Access to Healthcare for Uninsured Migrants in Germany: A Qualitative Study on the Involvement of Public Authorities in Nine Cities
Source: Int J Environ Res Public Health. 2022 May 28;19(11):6613. doi: 10.3390/ijerph19116613 (PMC9180213; doi:10.3390/ijerph19116613)

Supplementary File S3: Graphical presentation of networks mentioned during the interviews

For illustration of the research process, we provide a courtesy translation of these diagrams.

This visualisation was fabricated to aid individual case analysis during the research process and does not present a formal systematisation of care settings. Network connections are primarily pooled from the code category “NETZWERK”, and we only depict network connections that were mentioned during the interview. Therefore, underrepresentation of real-life interactions is very likely. Annotations to network connections are not systematic and are to facilitate reading of diagrams.

For illustration of the research process, we provide a courtesy translation of these diagrams.

Abbreviations: Local public health authority (LPHA), social security office (SSO), state public health authority (SPHA), interview person (P), care setting (S), non-governmental organisation (NGO), uninsured migrants (UM), tuberculosis (Tbc), Krankenversicherung (KV, health insurance), Human immunodeficiency virus (HIV), Infection Protection Act (“Infektionsschutzgesetz” IfSG).

Meaning of the indicators:

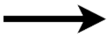 Network connection

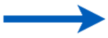 Outsourcing (Based on the Code “Outsourcing (Ehrenamtlich)”

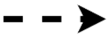 Planned connection

Thickness of arrows does not indicate stronger/weaker relations.

LPHA1 P1 S1

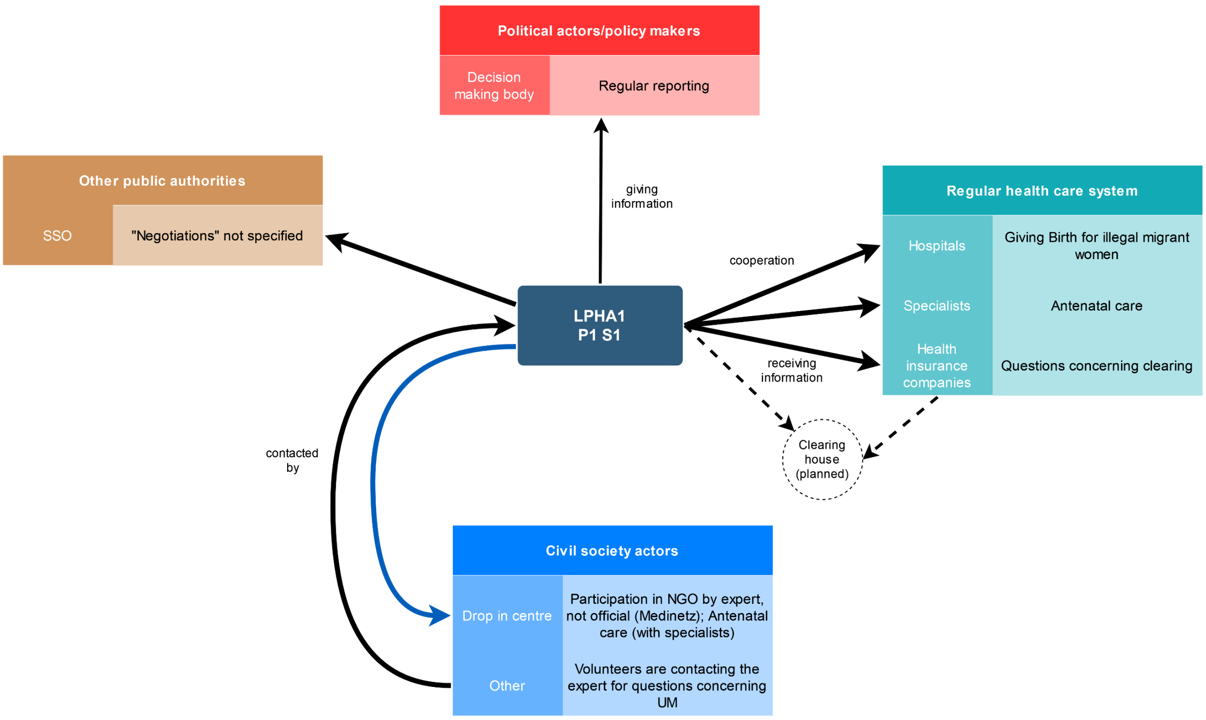

LPHA7 P10 S2

SSO2 P11 S2

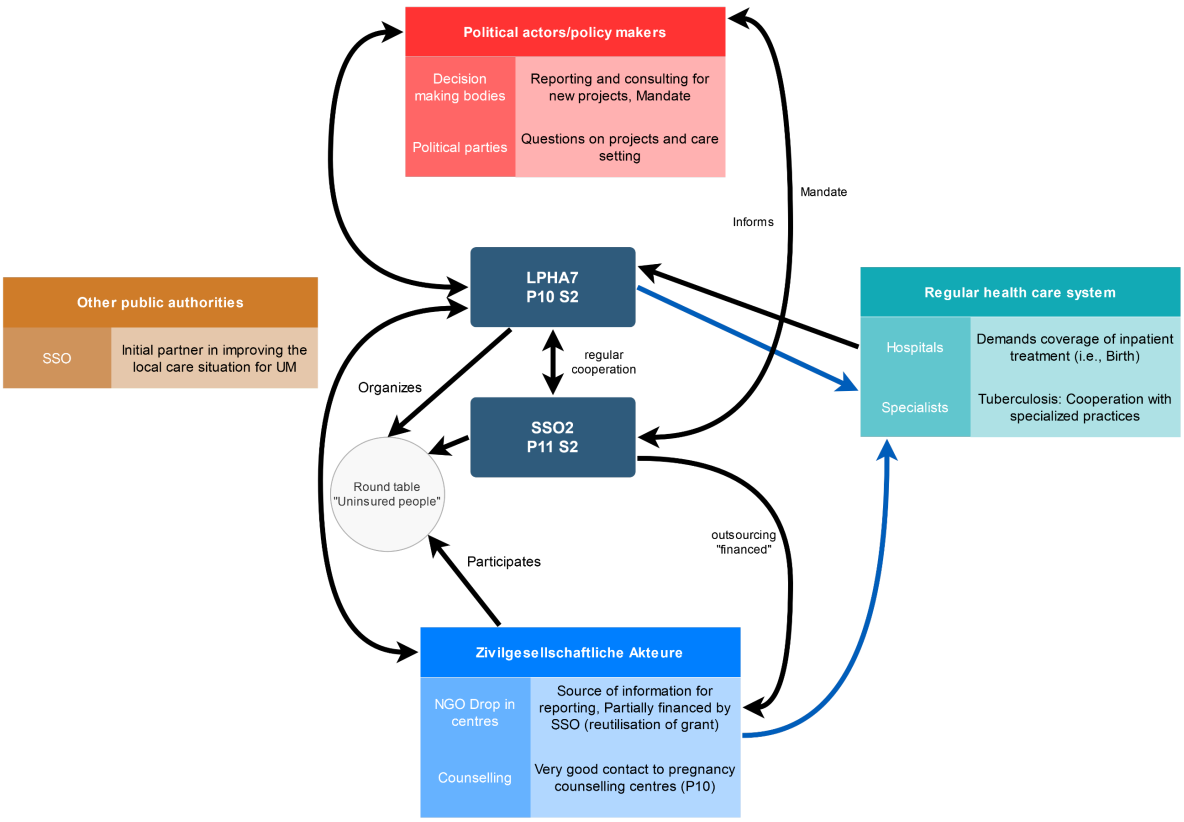

LPHA2 P2 S3

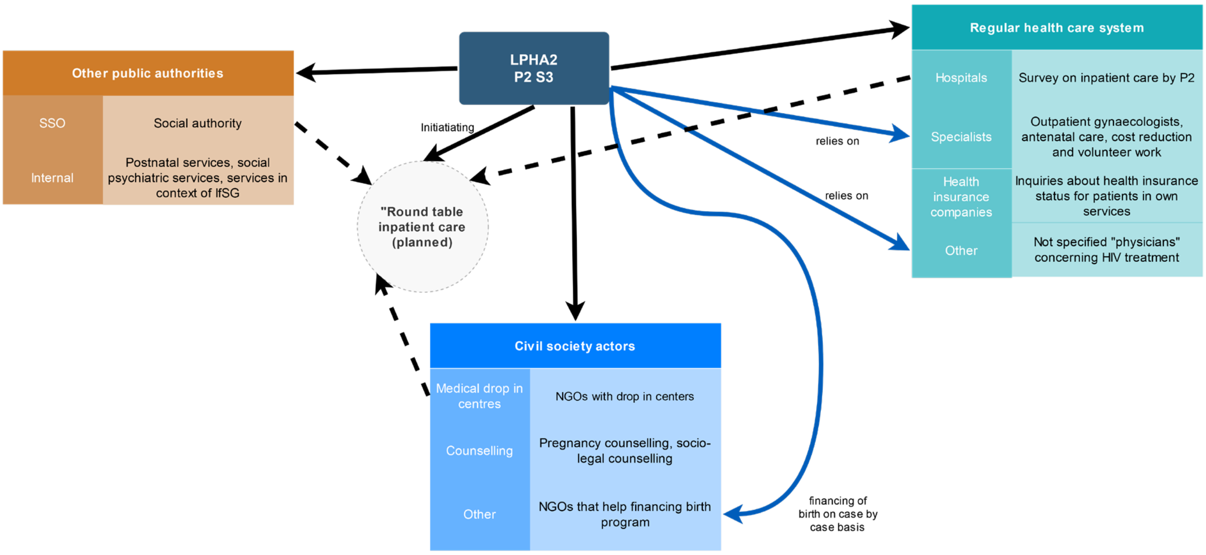

LPHA3 P3 S4

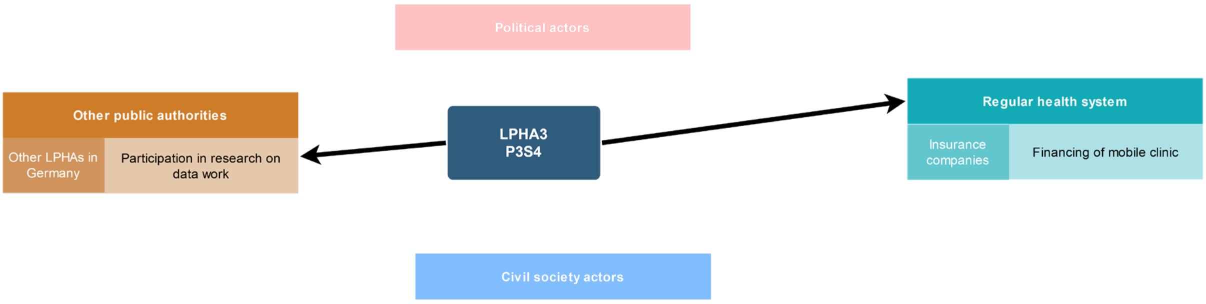

SSO3 P5 S5
LPHA4 P6 S5

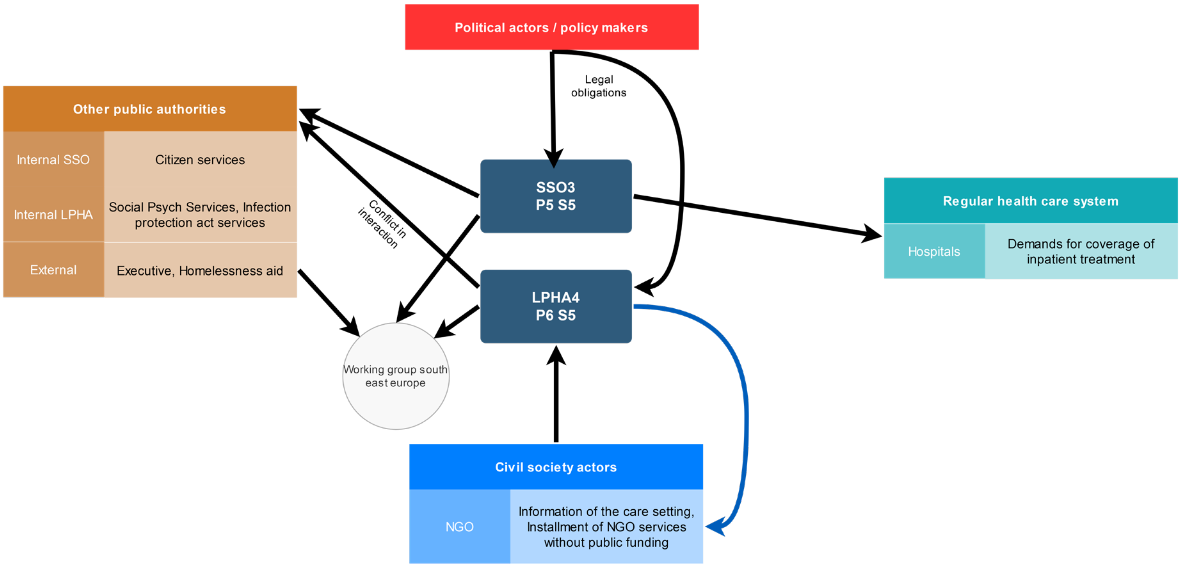

LPHA5 P8 S6

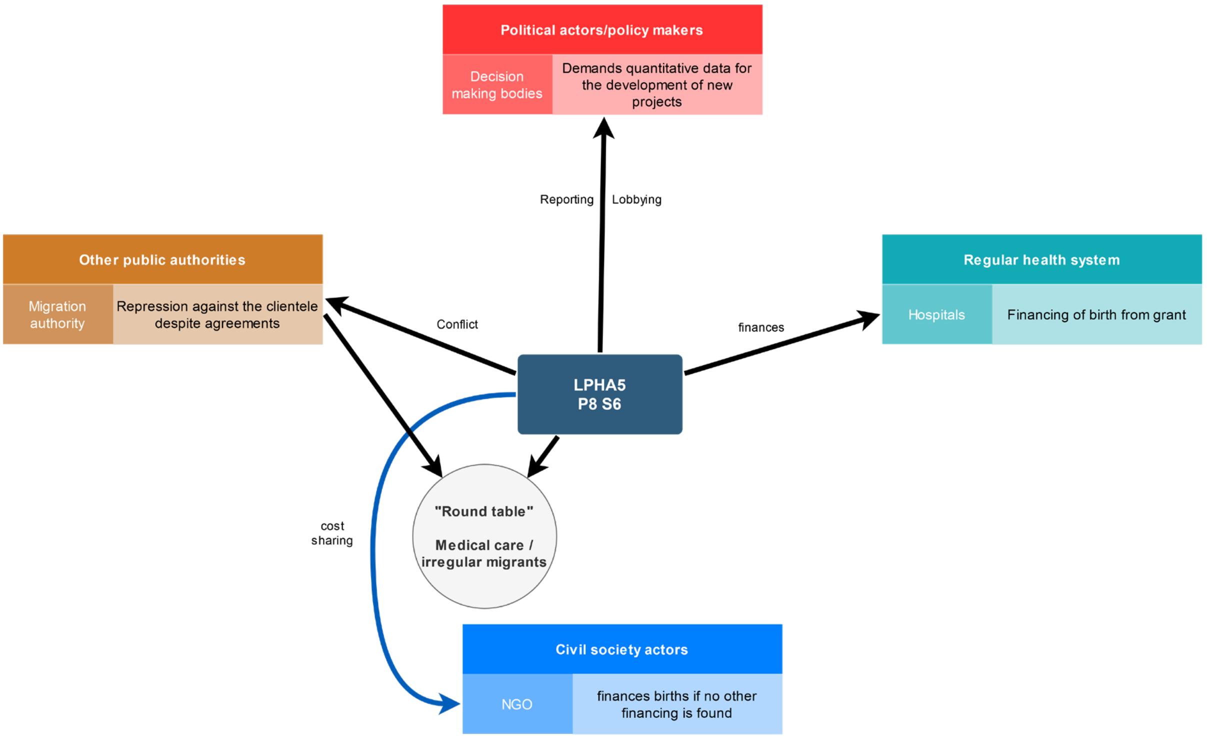

LPHA6 P9 S7

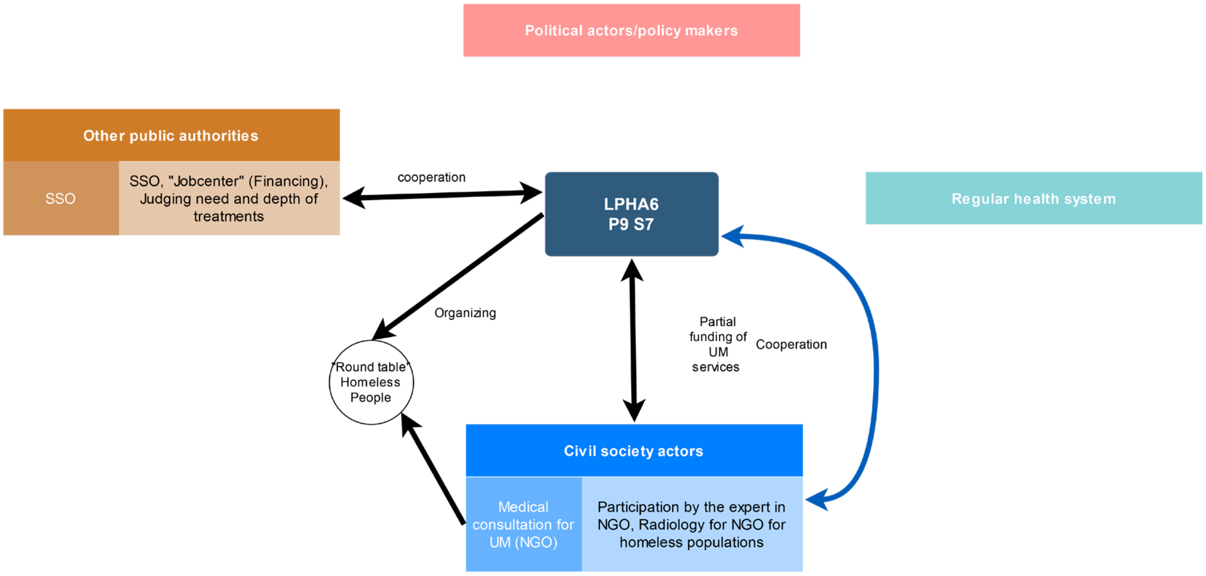

Political actors/policy makers
Other public authorities
SSO
SSO, "Jobcenter" (Financing), Judging need and depth of treatments
cooperation
LPHA6 P9 S7
Regular health system
Organizing
"Round table" Homeless People
Partial funding of UM services
Cooperation
Civil society actors
Medical consultation for UM (NGO)
Participation by the expert in NGO, Radiology for NGO for homeless populations

SSO1 P4 S8

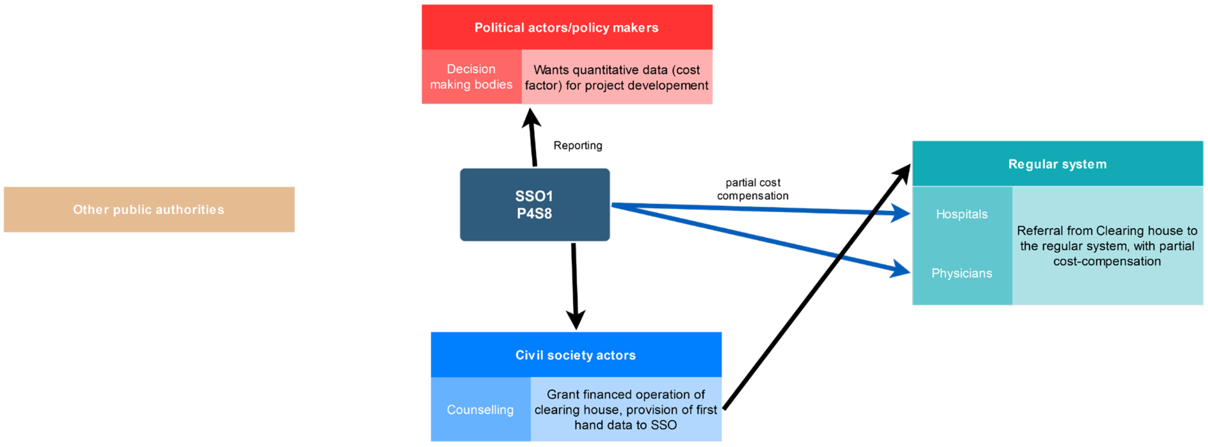

SPHA 1 P71 S9

SPHA 1 P72 S9

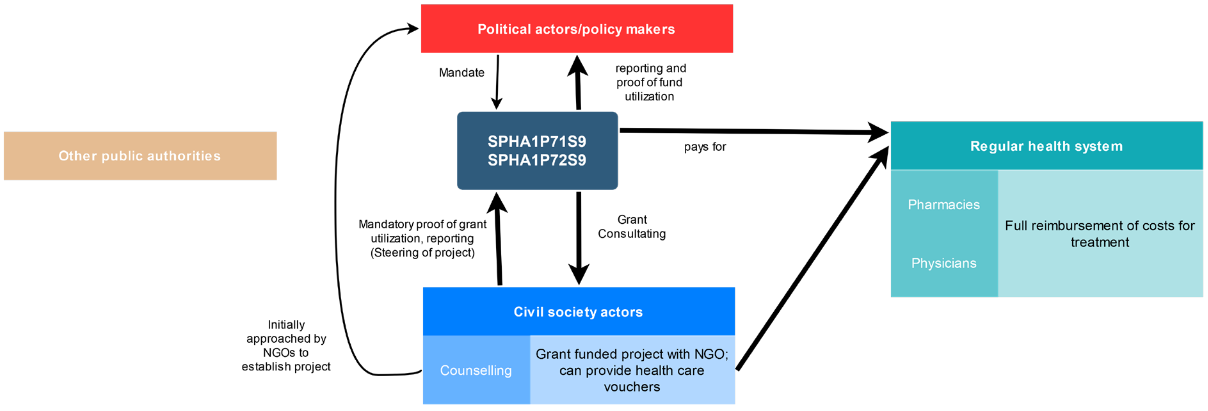

Supplement: Supplementary file 1 [file ijerph-19-06613-s001.zip › Supplementary File S3.pdf]
